# Advancements and future perspectives in nutrient film technique hydroponic system: a comprehensive review and bibliometric analysis

**DOI:** 10.3389/fpls.2024.1504792

**Published:** 2024-12-18

**Authors:** Onofrio Davide Palmitessa, Angelo Signore, Pietro Santamaria

**Affiliations:** Department of Soil, Plant and Food Sciences, University of Bari “Aldo Moro”, Bari, Italy

**Keywords:** water use efficiency, soilless, nutrient solution management, climate changes, environmental impact

## Abstract

In the context of climate change, reducing the environmental impact of agriculture has become increasingly critical. To ensure sustainable food production, it is essential to adopt cultivation techniques that maximize resource efficiency, particularly in water and nutrient usage. The Nutrient Film Technique (NFT) is one such hydroponic system, designed to optimize water and nutrient use, making it a valuable tool for sustainable agriculture. This bibliometric review examines the evolution of NFT research from 1977 to 2023, focusing on the growing interest in this method as a solution to the agricultural challenges posed by climate change. Through the analysis of 774 scientific documents, this review highlights an upward trend in NFT-related studies, with a noticeable shift from conference proceedings to peer-reviewed journal articles, particularly in recent years. *Acta Horticulturae* has been a leading journal in this field, underscoring the significance of early conference contributions. Lettuce and tomatoes have emerged as the primary crops studied in NFT systems, demonstrating the technique’s broad applicability. Research on lettuce has primarily focused on nitrate accumulation and biofortification, aiming to improve both the nutritional quality and safety of the crop. Studies on tomatoes have explored challenges related to oxygen concentration in the nutrient solution, where innovations such as the Nutrient Drip Technique (NDT) and the New Growing System (NGS) have shown promise in addressing these issues. Other key areas of NFT research include the effects of water salinity on crop growth and the integration of NFT with aquaponics systems, highlighting its potential for sustainable, water-efficient crop production. However, challenges such as nutrient imbalances and disease management persist. This review underscores the growing relevance of NFT in the pursuit of environmentally sustainable agriculture. Continued innovation and research are essential to optimizing nutrient management, refining environmental controls, and exploring new crop varieties, thereby enhancing the potential of NFT for sustainable farming systems.

## 1 Introduction

**Figure 1:**
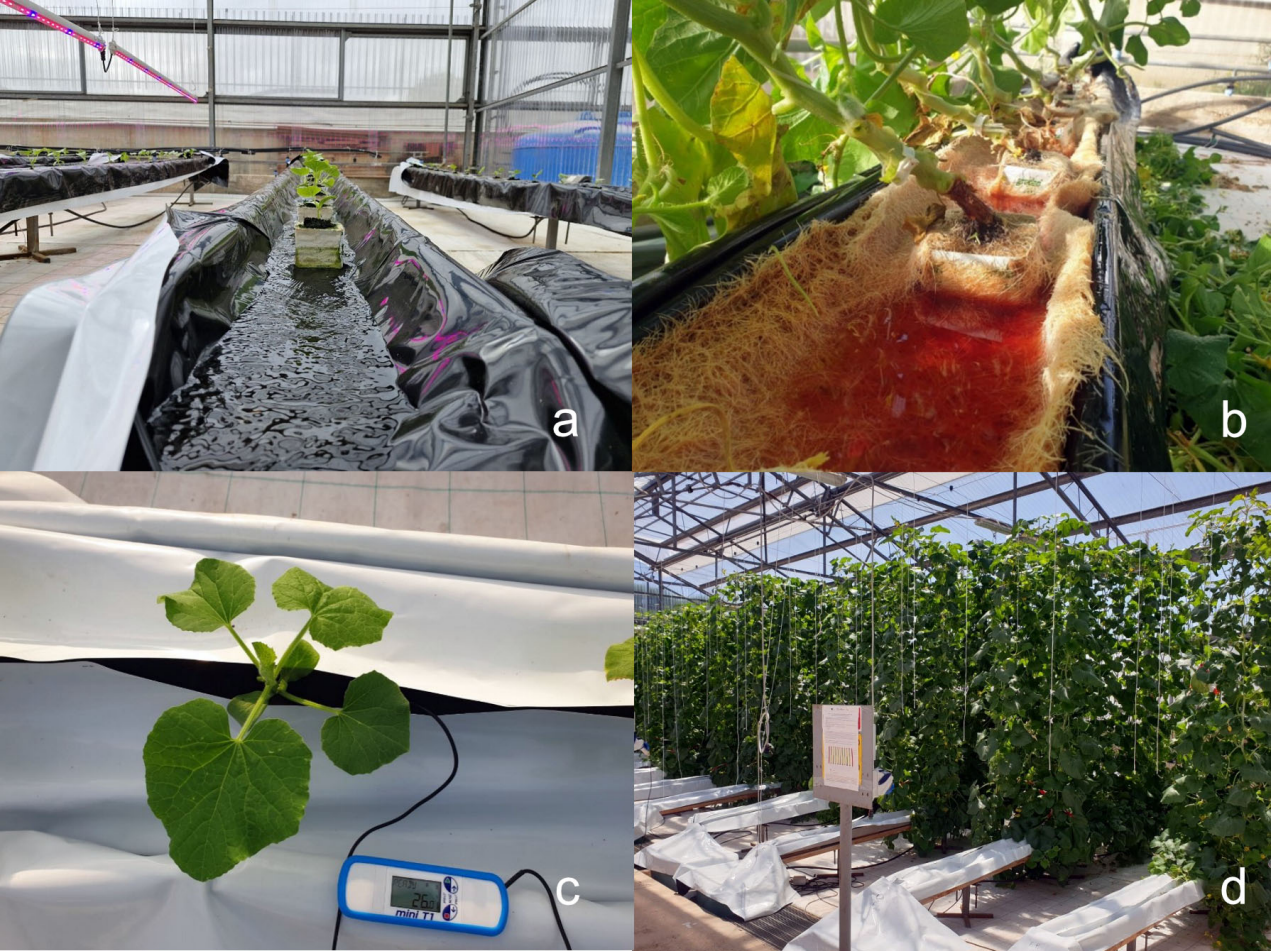
**(A)** NS flow in NFT cultivation system. **(B)** Excessive root development in unripe melon (*Cucumis melo* L.), landrace called ‘Scopatizzo’, grown in NFT, 80 days after transplant. **(C)** Continuous monitoring of root temperature in ‘Scopatizzo’ (*C. melo*) grown in NFT. **(D)** Overview of an unripe melon (Cucumis melo L.) landrace, called ‘Scopatizzo’, growth with NFT system and three levels of NaCl in the NS (0, 2.5 and 5 mM).

Climate change is significantly affecting agricultural activities by altering weather patterns, increasing the frequency of extreme events, and shifting growing seasons (Raza et al., 2019). These changes pose a global threat to crop yields and food security (Raj et al., 2022). Traditional farming is particularly vulnerable, as it relies heavily on stable environmental conditions (Owino et al., 2022). However, innovative agricultural practices such as greenhouse farming and soilless cultivation (hydroponics and aeroponics) offer promising solutions (Iglesias and Garrote, 2015). Greenhouses provide controlled environments, shielding crops from extreme weather and pests while allowing year-round production (Iglesias and Garrote, 2015). Soilless cultivation methods, particularly closed systems, minimize water usage and reduce dependency on fertile soil, making agriculture more resilient to climate change (Gebereegziher, 2023). These techniques not only boost productivity but also mitigate the adverse effects of climate variability on food supply. Soilless culture can be defined as “any method of growing plants without the use of soil as a rooting medium, in which the inorganic nutrients absorbed by the roots are supplied via the irrigation water” (Savvas et al., 2002). The first scientific description of crop production using the recirculating nutrient solution (NS) technique was made in 1929 by Gericke (1937). The general term “soilless culture” (SC), rather than “hydroponics,” is typically used to describe all soilless cultivation techniques. The use of the term “hydroponics” is not considered appropriate when organic substrates are involved (Park and Williams, 2024). However, many authors still use “hydroponics” as a synonym for “soilless culture” (Savvas et al., 2002). Soilless culture can be classified into "water culture" and "substrate culture" (Tüzel et al., 2019). The basic requirements for "water culture" include root aeration, darkness at root level, and plant support, while in "substrate culture," a porous organic or inorganic mass termed substrate or growing medium provides support to the plants, and the NS is delivered directly to the root zone (Tüzel et al., 2019). Water culture cultivation, particularly through systems such as the Nutrient Film Technique (NFT, **Figure 1A**), offers innovative solutions for sustainable agriculture (Samba et al., 2023). The NFT system delivers NS directly to plant roots, eliminating the need for growing media and reducing water and fertilizer usage as no nutrient solution is allowed to drain off the system. While NFT holds promises for a wide range of crops, its effectiveness varies depending on factors such as crop type, system layout, and management practices (Kumar and Singh, 2023). The NFT system was developed at the Glasshouse Crops Research Institute (Littlehampton, England) in the late 1960s, although similar methods had been used for research purposes at least a decade earlier (Graves, 1983). The NFT system offers several notable advantages over traditional soil-based cultivation methods (Burrage, 1999; Rajaseger, 2023). Unlike conventional farming, the NFT system does not require soil, eliminating soil-related issues such as erosion, compaction, and nutrient depletion (Graves, 1983). It also uses significantly less water compared to traditional farming methods, as water may be recirculated within the system, leading to savings in both water and fertilizers, making it an environmentally friendly option (Chan and Jerca, 2022). Additionally, the absence of soil reduces the risk of soil-borne diseases and pests, creating a healthier growing environment. The NFT system is space-efficient, making it suitable for urban farming and areas with limited land availability (Hosseini et al., 2021). Moreover, vertical NFT setups maximize space utilization, allowing for higher crop yields in compact environments (Hosseini et al., 2021). Plants in NFT systems also tend to grow faster than those in traditional farming methods, thanks to the direct delivery of nutrients to the roots and optimal root oxygenation within the nutrient film (Al-Maskri et al., 2010). Furthermore, in the NFT system, it is not necessary to precisely define the plants' water needs to adjust the water supply between irrigation cycles. This is because the system provides a continuous or intermittent thin film of nutrient-rich water that flows over the roots. With constant access to water and nutrients, plants are not at significant risk of water stress between irrigation events (Treftz and Omaye, 2016). However, despite its numerous advantages, the NFT system comes with challenges and considerations. While theoretically capable of supporting a wide range of crops, it is most effective for short-term crops with growth cycles ranging between 30 and 50 days (Burrage, 1993). Longer-term crops may encounter difficulties as their root systems expand and fill the narrow channels (**Figure 1B**), potentially leading to nutrient and oxygen deficiencies (Suhl et al., 2019). The limited volume of NS in the NFT system makes it susceptible to temperature fluctuations (**Figure 1C**), particularly in climates with significant temperature variations (Wang et al., 2022). The NFT system also has limited buffering capacity in case of interruptions in water and nutrient supply. Any shortage, such as power outages or pump failures, can quickly impact plant health and crop yield. Therefore, adequate backup systems and contingency plans are essential to mitigate these risks. Additionally, the close proximity of plant roots in NFT channels increases the risk of disease transmission, as root-borne pathogens can rapidly spread throughout the system, compromising plant health and productivity. To minimize this risk, strict sanitation protocols and proactive disease management strategies are necessary. In a recent study conducted by Palmitessa et al. (2024), it was demonstrated that a closed NS cycle in NFT systems can be a viable method for utilizing moderately saline water (with a NaCl concentration of up to 5 mM) to enhance the cultivation of a local Apulian landrace of immature melon (*Cucumis melo* L.), called ‘Scopatizzo’ (Palmitessa et al., 2021; Somma et al., 2021; **Figure 1D**). The results showed that the NFT system achieved a water use efficiency (WUE) of up to 25 L/kg of fruit produced. This finding is particularly significant, as the average WUE for cucumber production in soilless systems typically ranges from 50 to 100 L/kg of fruit (Savvas and Gruda, 2018). Improving WUE in agriculture is crucial for sustaining food production in the face of growing water scarcity, reducing environmental impact, and supporting climate change adaptation efforts. Efficient water management ensures that crops receive the optimal amount of water, minimizing waste and enhancing yield. According to the FAO, improving water efficiency can also alleviate pressure on freshwater resources (FAO, 2017). However, fluctuations in pH, dissolved oxygen, and temperature of the NS, as well as root development in the channels, have made the management of the entire cultivation system challenging (Palmitessa et al., 2024). Building on the aspects discussed above, the aim of this bibliometric review is to explore how studies and applications of NFT have been approached by the scientific community over the years. Additionally, this review seeks to gather useful insights to identify the main themes addressed in research utilizing this cultivation technique, with the goal of defining its full potential. Consequently, compiling a review of the key scientific contributions involving this technique is highly valuable for assessing the current state of the art and identifying future prospects for enhancing its application in both commercial and research contexts.

## 2 Materials and methods

A comprehensive search was conducted in April 2024 using the two main databases in the agricultural field, namely Scopus and ISI Web of Science (referred to as WoS hereafter). The query focused on the NFT soilless cultivation technique. Given the ambiguity of the acronym "NFT”, boolean operators were employed in both search engines to exclude irrelevant terms. The queries, conducted in english, were as follows:

- Scopus: (TITLE-ABS-KEY ("NFT") OR TITLE-ABS-KEY ({Nutrient-film-technique}) OR TITLE-ABS-KEY ({Nutrient-film technique}) OR TITLE-ABS-KEY ({Water film technique}) OR TITLE-ABS-KEY ({Water-film technique}) AND NOT TITLE-ABS-KEY ({fungible}) AND NOT TITLE-ABS-KEY ({animal}) AND NOT TITLE-ABS-KEY ({neurofibrillary}) AND NOT TITLE-ABS-KEY ({Nitrofurantoin}) AND NOT TITLE-ABS-KEY ({near freezing})) AND (LIMIT-TO (SUBJAREA, "AGRI")) AND (EXCLUDE (DOCTYPE, "ed") OR EXCLUDE (DOCTYPE, "no")) AND (LIMIT-TO (LANGUAGE, "English"))1
- WoS: Nutrient film technique (All Fields) not fungible (All Fields) not bacteria (All Fields).

In addition, WoS allows to generate a permalink to the performed query: https://www.webofscience.com/wos/woscc/summary/a7ce2512-bfe1-4939-ba09-c0f2fe4273e1-0105e70830/relevance/1 (last access 20/09/2024).

After completing the search, we exported the results in.bib format from Scopus and as plain text (.txt) from WoS. For our analysis, we used two different software tools: Bibliometrix and Orange Data Mining. Bibliometrix is an open-source tool, provided as an R package (Aria and Cuccurullo, 2017), for performing quantitative research in scientometrics and bibliometrics. Specifically, we worked with Biblioshiny, a web app that offers a user-friendly interface for Bibliometrix, making it accessible to researchers without coding skills (https://www.bibliometrix.org/home/index.php/layout/biblioshiny). Orange (https://orangedatamining.com – last accessed on 30/09/2024) is an open-source software used for data mining, data visualization, and machine learning, which operates through visual programming (using various widgets) to create workflows.

**Figure 2:**
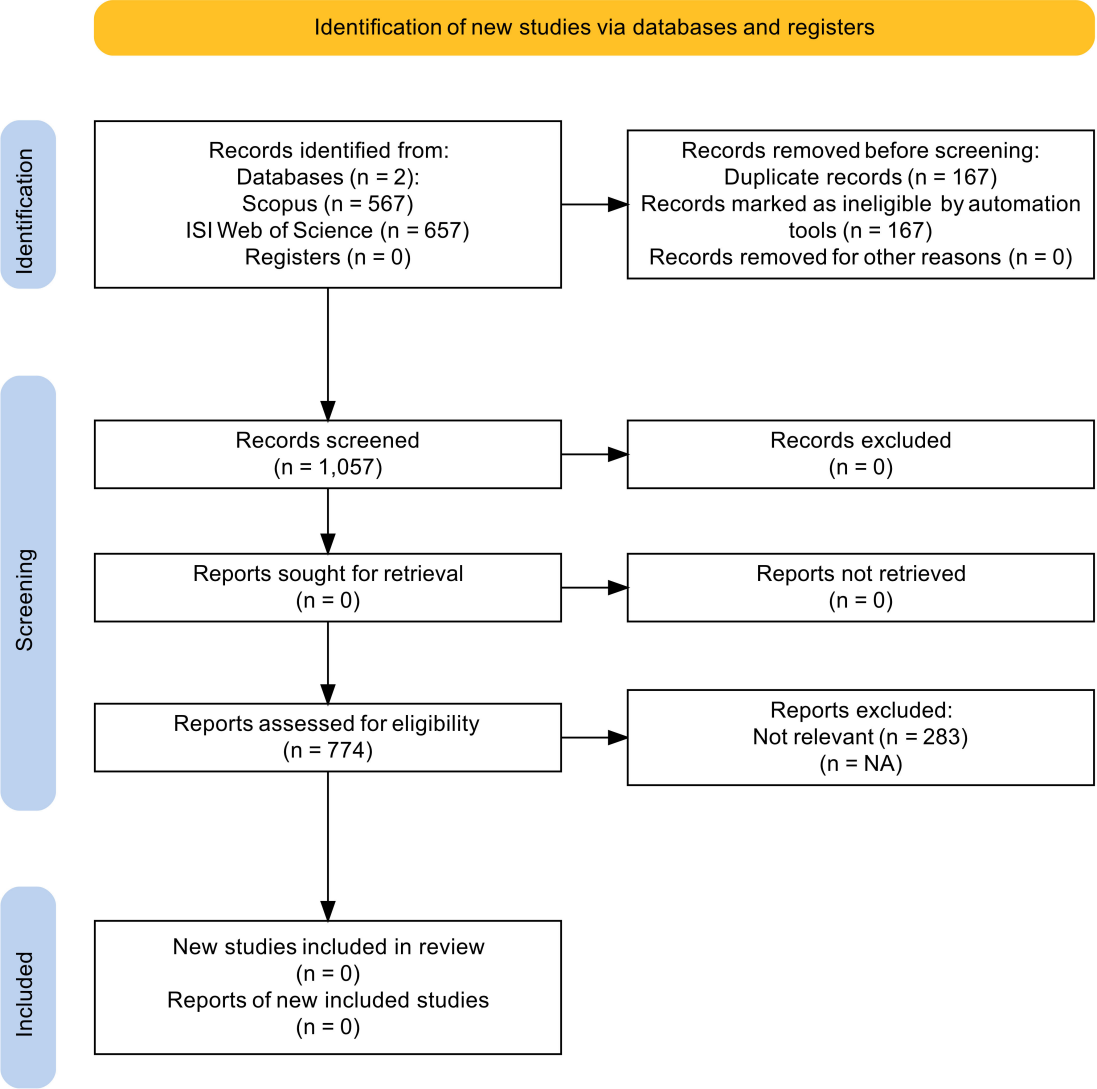
The PRISMA flowchart.

The Bibliometrix package identified and removed a total of 162 duplicate articles, leaving a dataset of 1057 records in an.xlsx file. However, to ensure no irrelevant records were included, we manually reviewed the titles and abstracts of all articles. This manual check identified 283 irrelevant articles, which were subsequently removed, resulting in a final dataset of 774 records. The complete PRISMA workflow (Haddaway et al., 2022) is depicted in **Figure 2**.

At this stage, we uploaded the final data file into Biblioshiny. Since the timespan of the articles ranged from 1977 to 2024, we chose to display only the articles published up to 2023, as 2024 is still ongoing. This resulted in a total of 752 records.

For a more detailed description of the Bibliometrix/Biblioshiny workflow, please refer to He et al. (2022).

## 3 Results

The first scientific work regarding the NFT topic was published in 1977 by Cooper and Charlesworth (1977). In their work, the authors were able to handle the nutrition in beefsteak tomato cultivated in NFT by means of pH and NS management.

**Figure 3:**
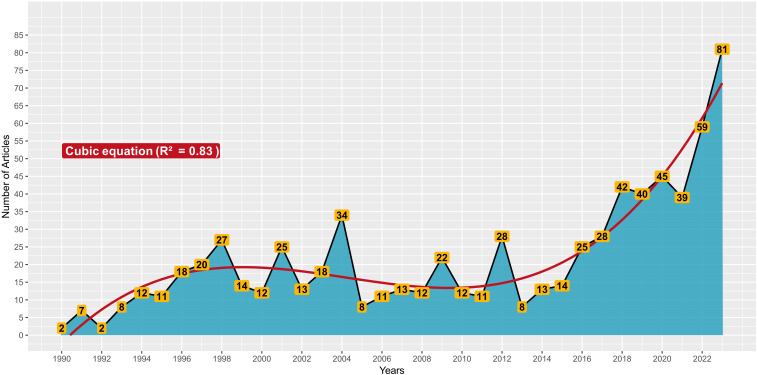
Cumulative production (on an annual basis – timespan: 1990-2023) of scientific papers on NFT.

The production of scientific papers reached a peak of 81 in 2023, with a steady increase that became a more stable trend starting from 2013 (**Figure 3**). As can be seen from **Figure 3**, until the early 1990s, only a few scientific papers were published on the NFT topic, while from 1991 onwards the scientific productivity on NFT technique steadily increased, showing peaks of productivity in 1998, 2001, and 2004, with 27, 25, and 34 scientific papers, respectively. Between 2005 and 2015, scientific publications regarding NFT were consistently below 20 papers per year, except for the years 2009 and 2012, in which 22 and 28 scientific publications were produced, respectively (**Figure 3**). From 2013 onwards, there was a continuous increase in scientific publications related to the NFT theme; particularly, comparing the scientific production in 2022 and 2023 with respect to 2021, it was 50% higher in 2022, and double in 2023 (**Figure 3**).

To better understand the "significance" or "spread" of scientific studies on NFT, it is important to frame the evolution of scientific publications concerning the entire area of interest of soilless cultivation. While in the 1990 – 1999 timespan NFT publications represented 1% of the "soilless" ones, in 2023, approximately 5% of publications on the topic of "soilless" concern the NFT technique (**Figure 3**). This increase can be attributed to advancements in knowledge and technology, which have made managing this cultivation technique less problematic than in the past. Furthermore, both for the "soilless" and "NFT" topics, the annual trend of scientific productivity from the 1990s to 2023 follows two different curves described by third-degree equations with coefficients of determination equal to 0.98 (Figure not shown) and 0.83 (**Figure 3**), respectively.

However, soilless systems encompass several cultivation methods, with NFT being just one of them. To highlight how the NFT trend has evolved compared to soilless systems in general, we investigated the percentage of scientific papers regarding NFT relative to the term "soilless". Furthermore, to gain a deeper understanding of the trend, we examined how scientific interest has changed over the years.

**Figure 4:**
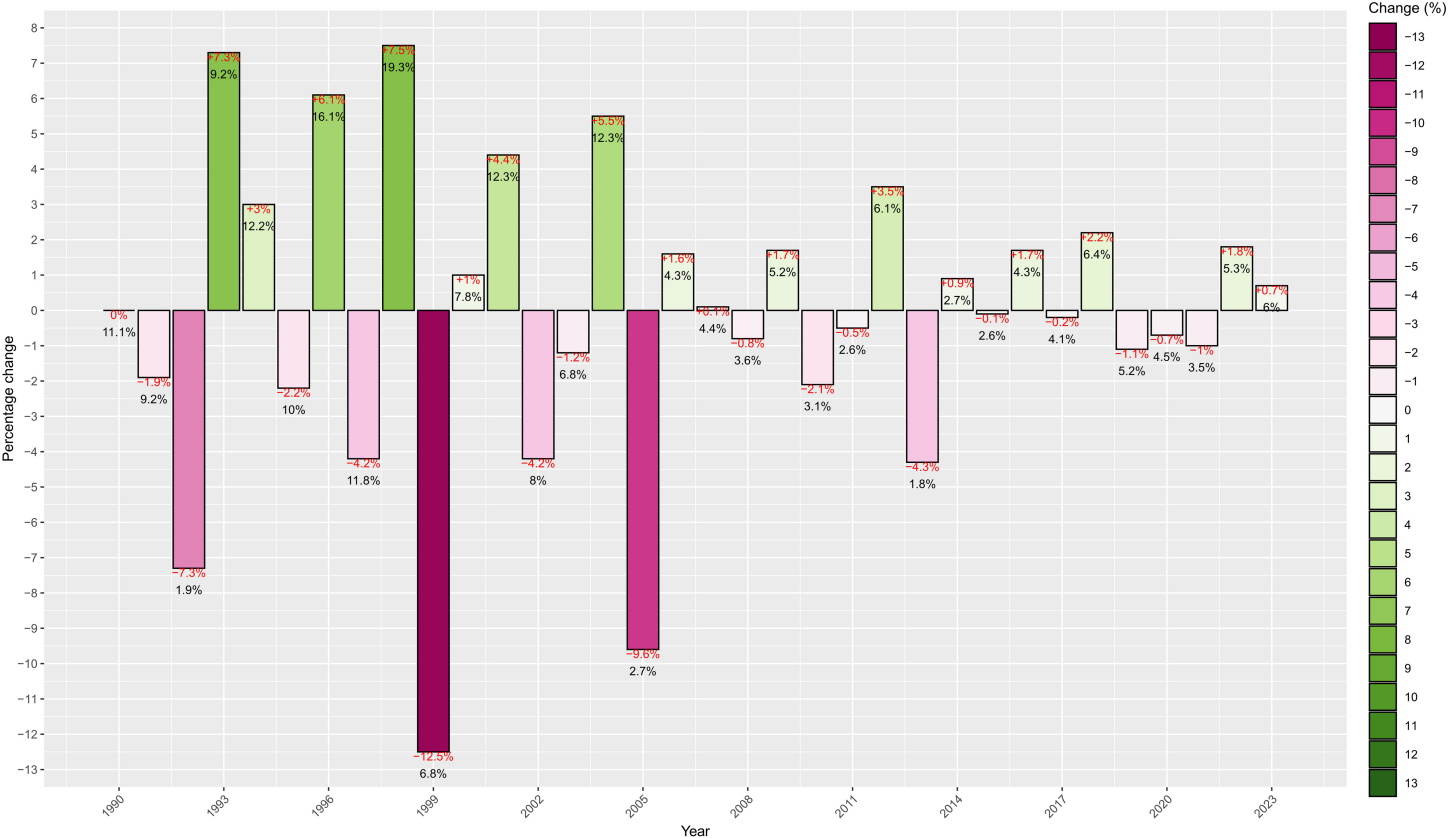
Percentage of scientific papers on NFT relative to soilless systems (black numbers) and percentage change from the previous year (red numbers and histogram values).

**Figure 4**, covering the timespan from 1990 to 2023 (before that year, very few pioneering articles were written), we analyzed the percentage of “NFT” compared to the more generic “soilless” term in articles and observed how this percentage varied with respect to the previous year.

From the analysis of **Figure 4**, the 1990-2023 period can be roughly divided into three intervals: until 1999, the “NFT” topic exhibited fluctuating interest, with some years experiencing significant decreases in percentage. In the second period (up to 2005), the negative percentage, indicating that fewer “NFT” articles were published compared to “soilless” ones, was less pronounced than in the first period, a trend that continued in the latter period (up to 2023).

**Figure 5:**
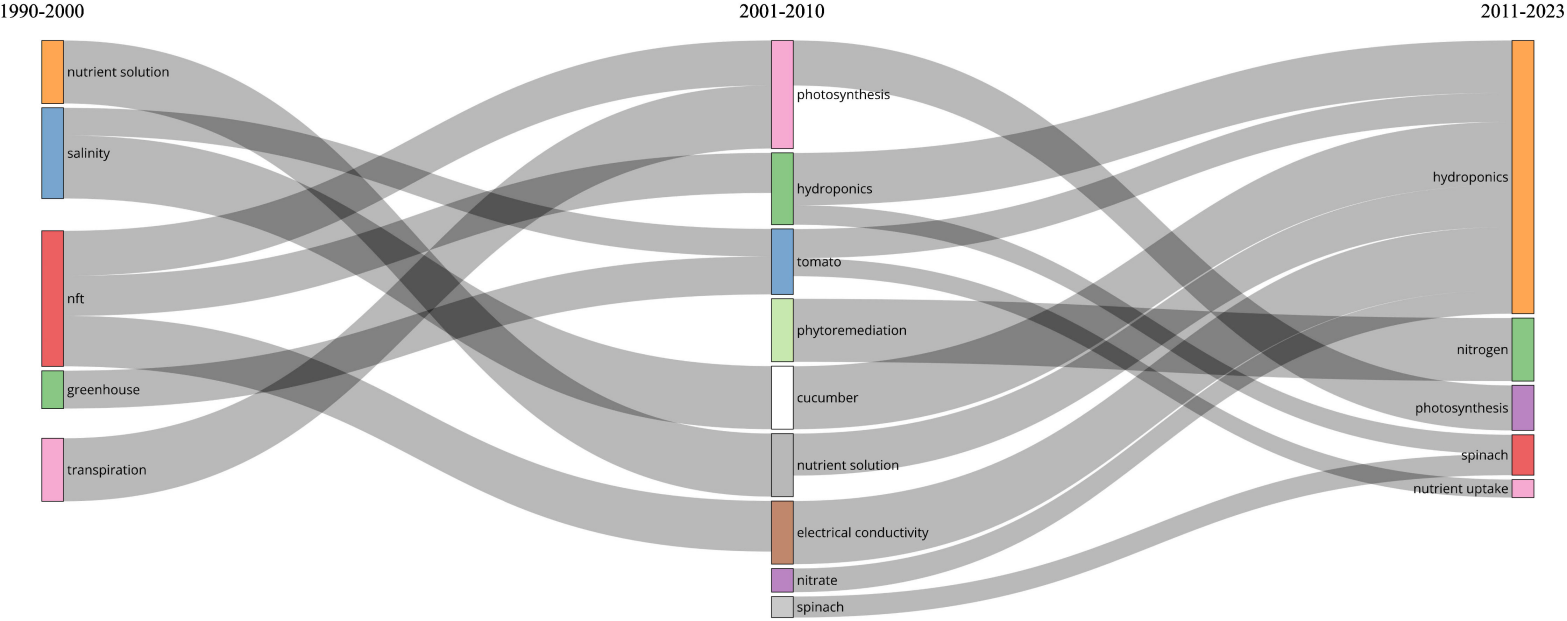
Thematic evolution of Authors' keywords over the 1990-2023 timespan.

Another interesting aspect relates to the authors' keywords (**Figure 5**). In the first ten years (1990-2000), research focused on the basic aspects of cultivation using the NFT method, such as NS, salinity, and greenhouse cultivation. In the second period (from 2001 to 2010, as shown in **Figure 5**), NFT was used to grow important crops like cucumber, tomato, and spinach, while also studying physiological aspects such as nitrate nutrition and photosynthesis, along with innovative applications like phytoremediation.

In recent years, several topics have been encompassed within the broader term “hydroponic,” indicating that the hydroponic cultivation system is more significant than individual topics (e.g., cucumber, electrical conductivity) within it. Another important point to highlight is that the term “tomato” in the 2001-2010 period merged with “nutrient uptake” (**Figure 5**), underscoring the importance of nutrition in producing high-quality yields for this essential crop.

**Figure 6:**
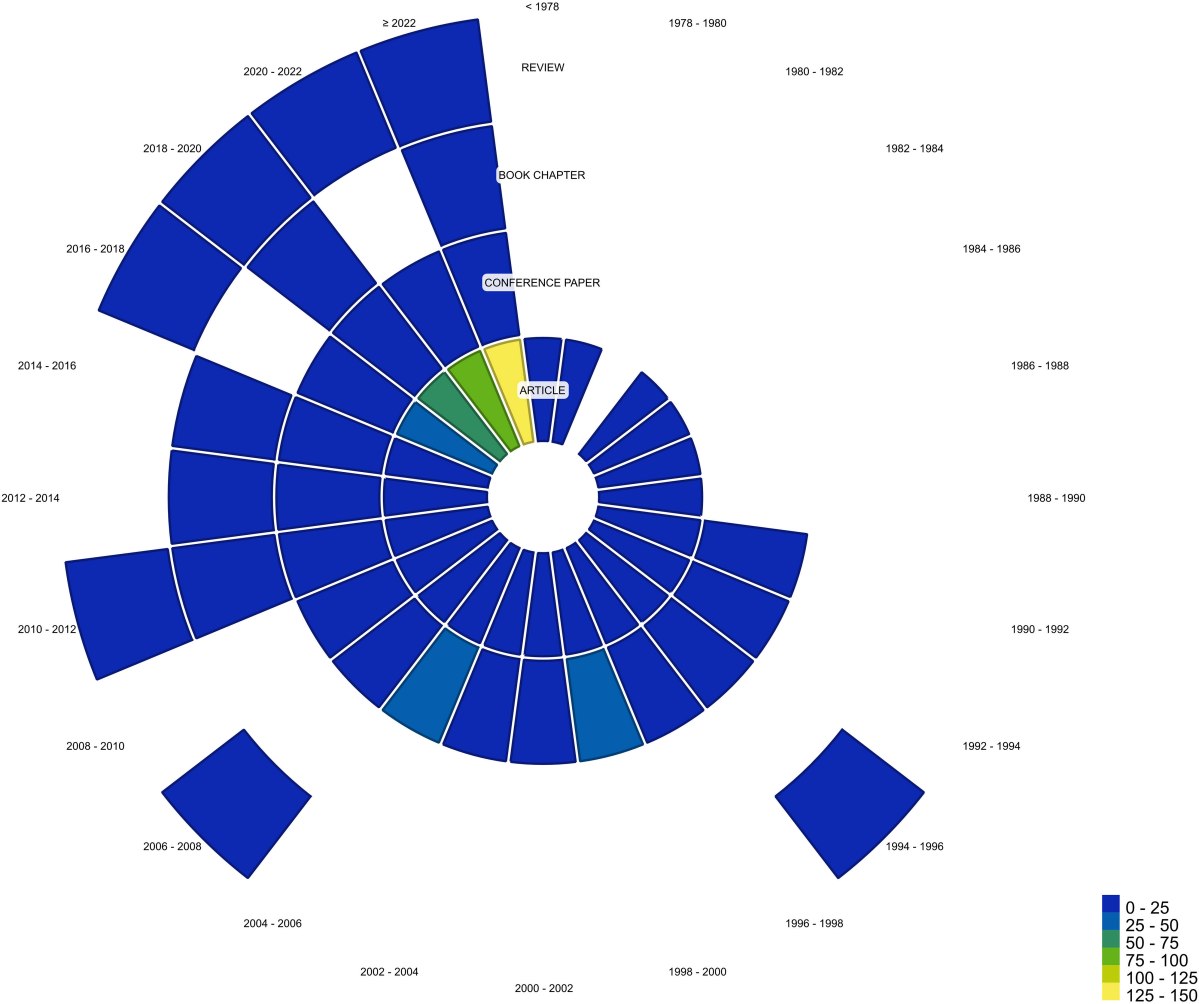
Number of scientific papers categorized by type and year.

The typology of scientific papers has changed over the years. "Scientific articles" have contributed most significantly to scientific advances, particularly between the 1990s and 2000s. The topic of NFT was primarily introduced to the scientific community through "conference papers," with the first such papers appearing by the end of the 1990s (**Figure 6**). Book chapters, with only seven publications, constituted the minority typology, with their publication beginning in 2010 (**Figure 6**).

From the mid-1990s until 2010, conference papers also played a significant role, with nearly 150 papers published—just a couple of dozen fewer than the number of articles during the same period (**Figure 6**). Reviews began to emerge in the mid-1990s, although continuous publication only started less than ten years ago. Book chapters did not enter the literature until 2010 (**Figure 6**).

**Figure 7:**
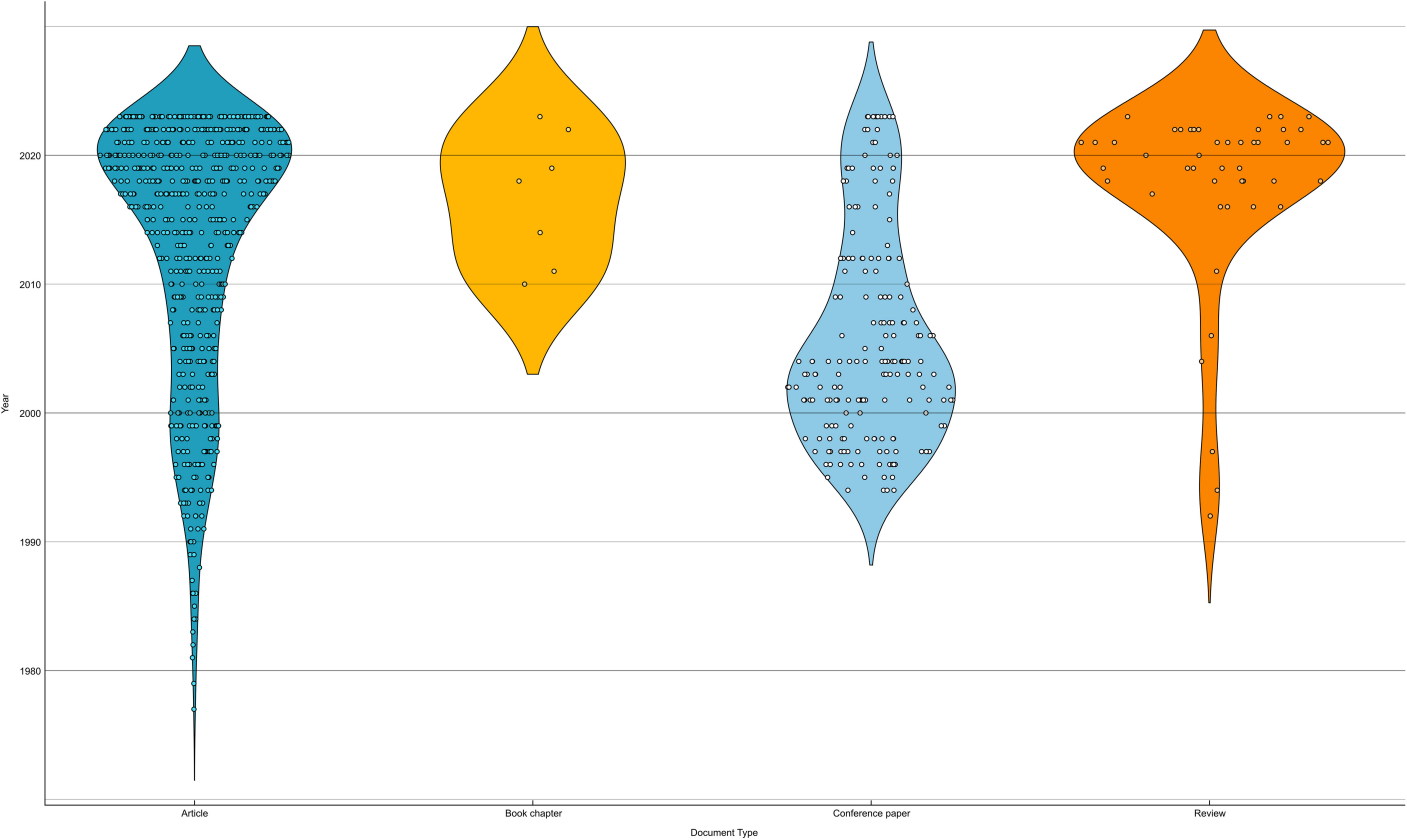
Scientific papers categorized by type and year (timespan: 1977-2023). Each dot in the violin plot represents an entry based on its corresponding document type.

In recent years, there has been a resurgence in discussions about NFT in "scientific articles" and "reviews". For the latter type, particularly in the last seven years, over 80% of the total production has been recorded, indicating a growing interest in both the NFT topic and the review-type content (**Figure 7**).

**Figure 8:**
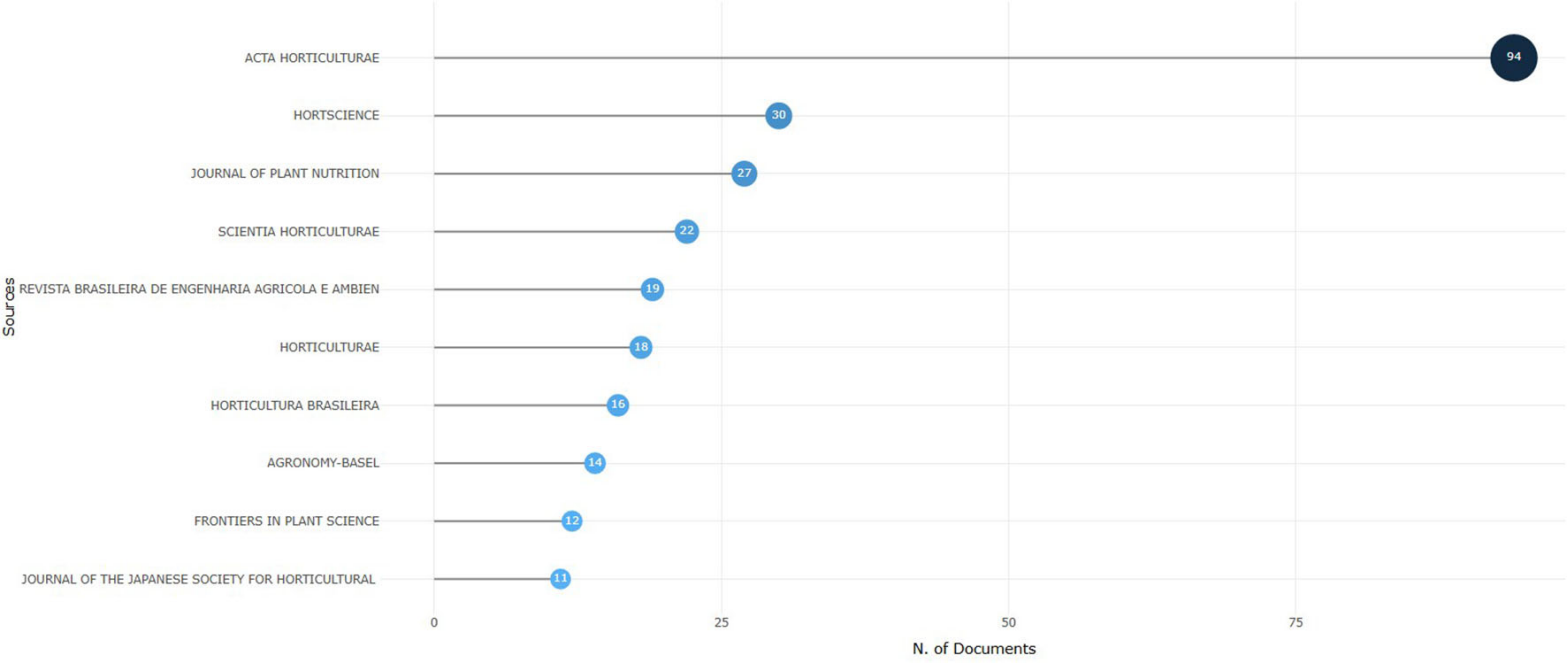
Most important journals according to the number of publications on NFT papers.

Currently, "Acta Horticulturae," with 94 papers, is the scientific journal that has published the highest number of contributions on NFT. This figure is 64, 67, and 72 papers more than those published in “HortScience,” “Journal of Plant Nutrition,” and “Scientia Horticulturae,” respectively (**Figure 8**). The high number of publications in “Acta Horticulturae” is mainly due to the production of conference papers, which, as shown in **Figure 7**, represented the primary avenue for scientific article publication until the early 2000s.

**Figure 9:**
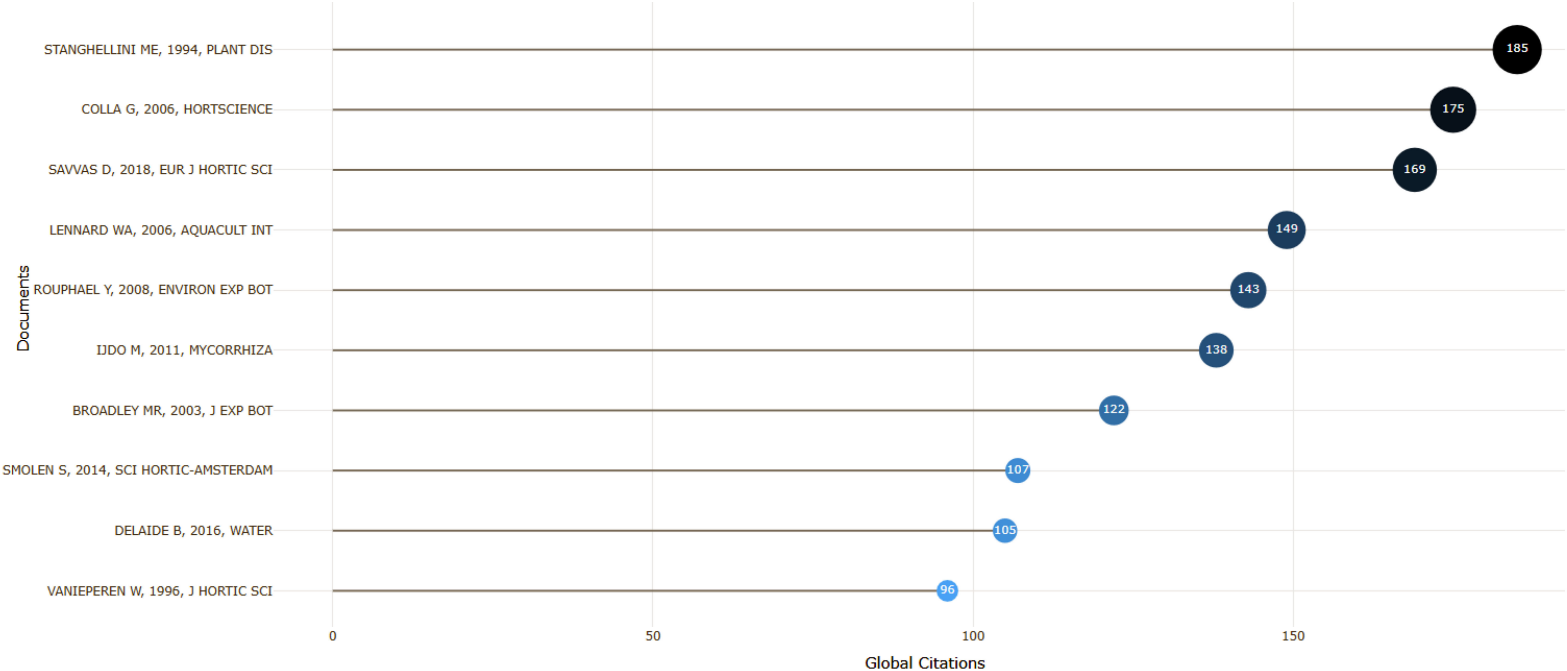
Most global cited Authors and documents.

Considering all the works published on NFT, the most globally cited documents, in descending order of publications, are: (Stanghellini and Rasmussen, 1994; Colla et al., 2006; Lennard and Leonard, 2006; Rouphael et al., 2008; Savvas and Gruda, 2018, IJdo et al., 2011, Broadley et al., 2003, Smoleń et al., 2014; Delaide et al., 2016 and Van Ieperen, 1996; **Figure 9**).

**Figure 10:**
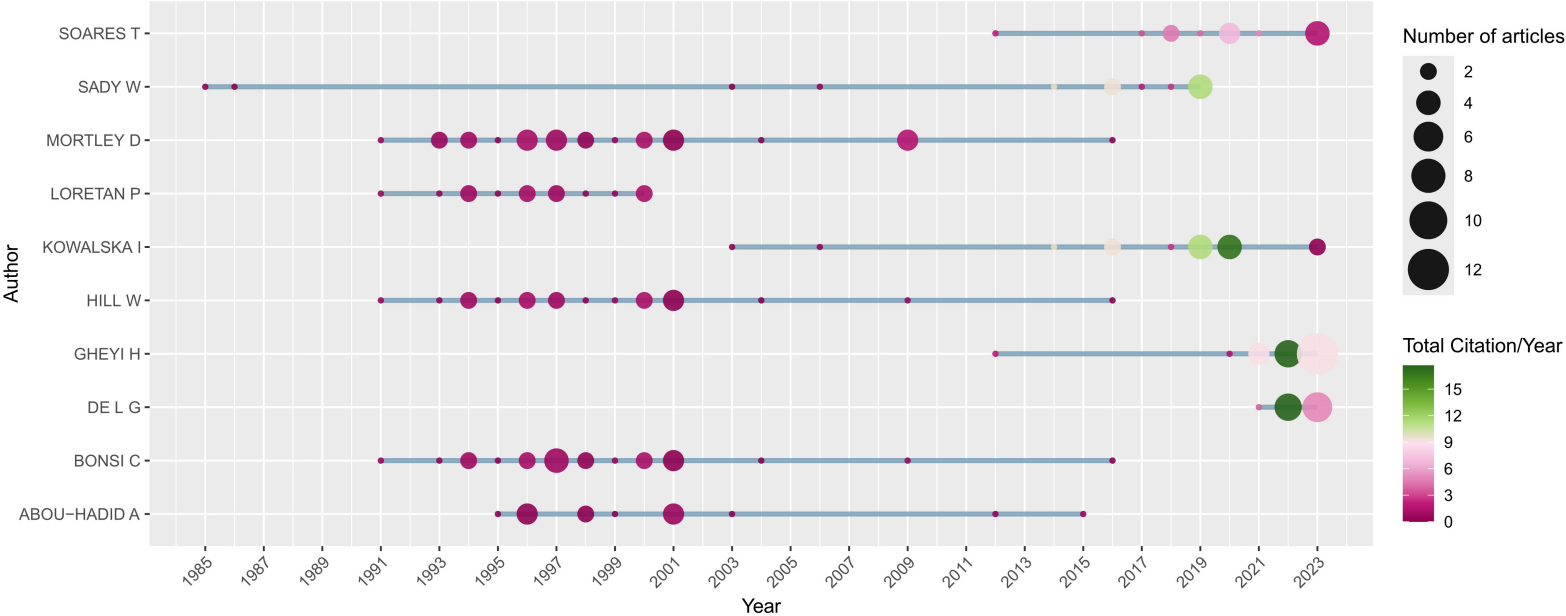
Authors’ scientific production across the specified timespan period.

In terms of scientific production broken down by author, Mortley, with 25 papers, is the scientist who has produced the most publications on NFT and has also had one of the longest spans of publication, ranging from 1991 to 2017 (**Figure 10**). Following him are Bonsi, Ghey, Hill, Kowalska, Abou Hadid, Loretan, Sady, Soares, and De, who have produced 22, 22, 19, 16, 13, 13, 13, 13, and 12 scientific works, respectively (**Figure 10**).

**Figure 11:**
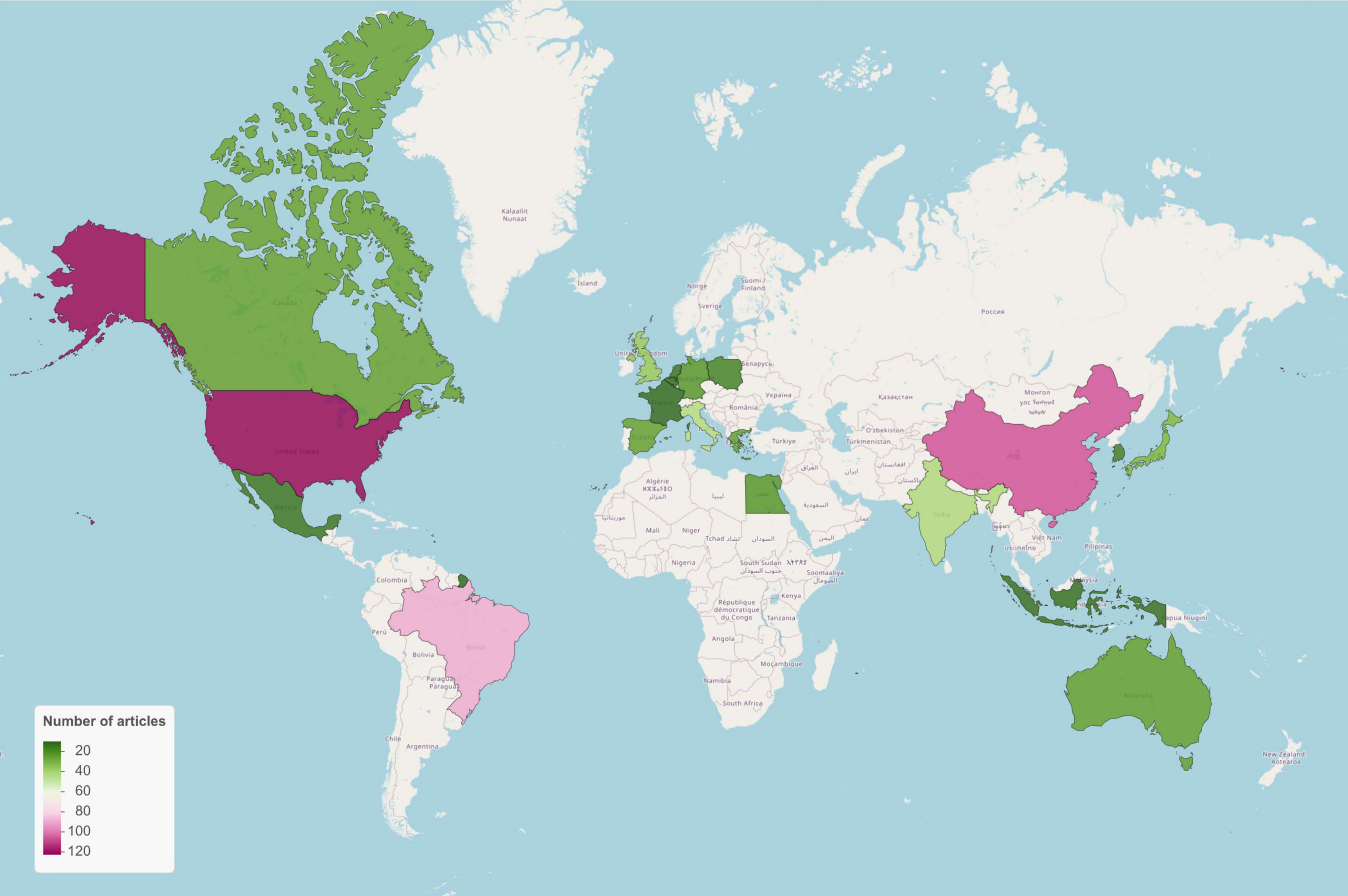
Number of publications on the NFT topic, categorized by country.

**Figure 12:**
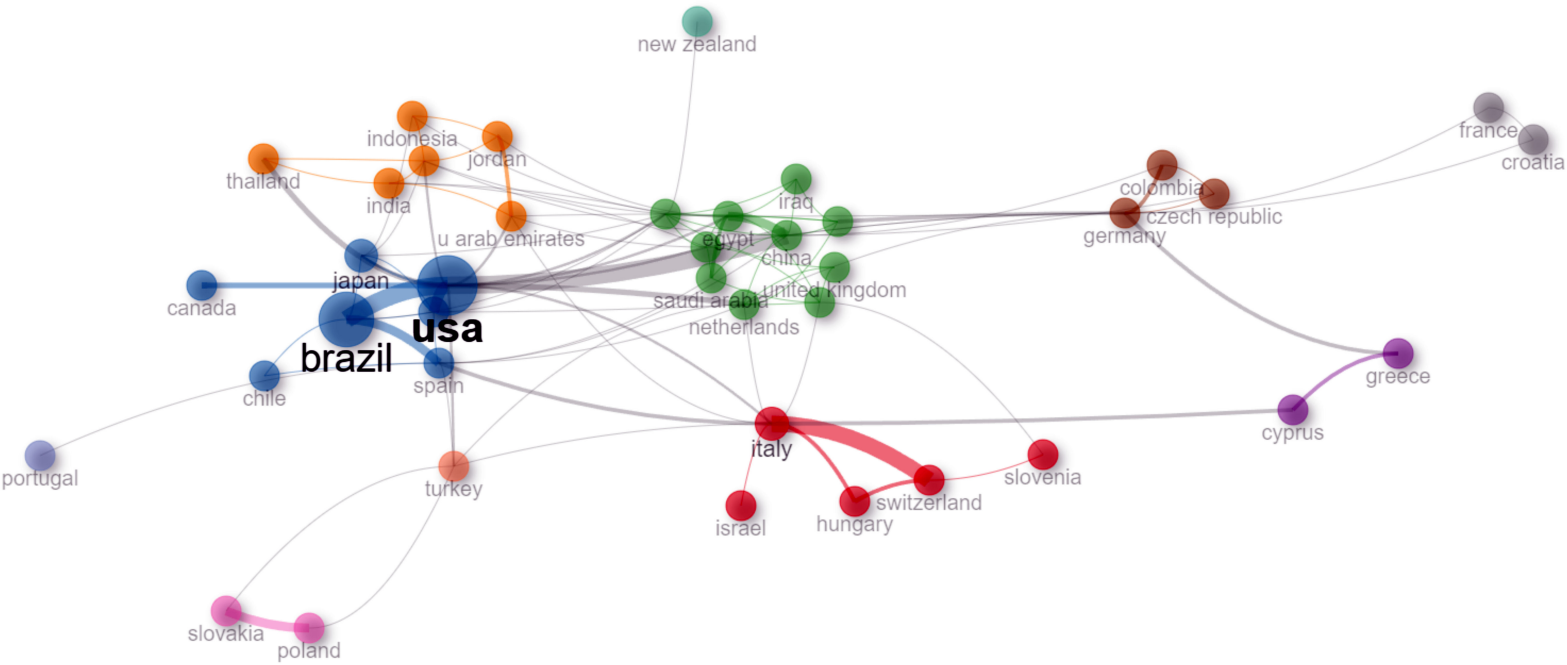
Collaboration network among countries for articles on the NFT topic.

At the national level, Brazil and the USA lead in the number of publications on the NFT topic, with 106 each (**Figure 11**). Following them by more than 70 publications, in decreasing order, are Japan, Italy, India, Poland, Egypt, and Greece (**Figure 11**). All other countries contribute fewer than 20 publications on the topic of NFTs (**Figure 11**). Brazil, the USA, and Japan are distinguished by their low percentage of scientific papers published in collaboration with other countries (less than 10%), while China, Belgium, and Italy have collaboration rates of 70%, 40%, and 35%, respectively (data not shown). Among the most frequent collaborations between countries are Brazil with the USA, Brazil with Spain, the USA with China, Italy with Switzerland, and China with Egypt (**Figure 12**).

**Figure 13:**
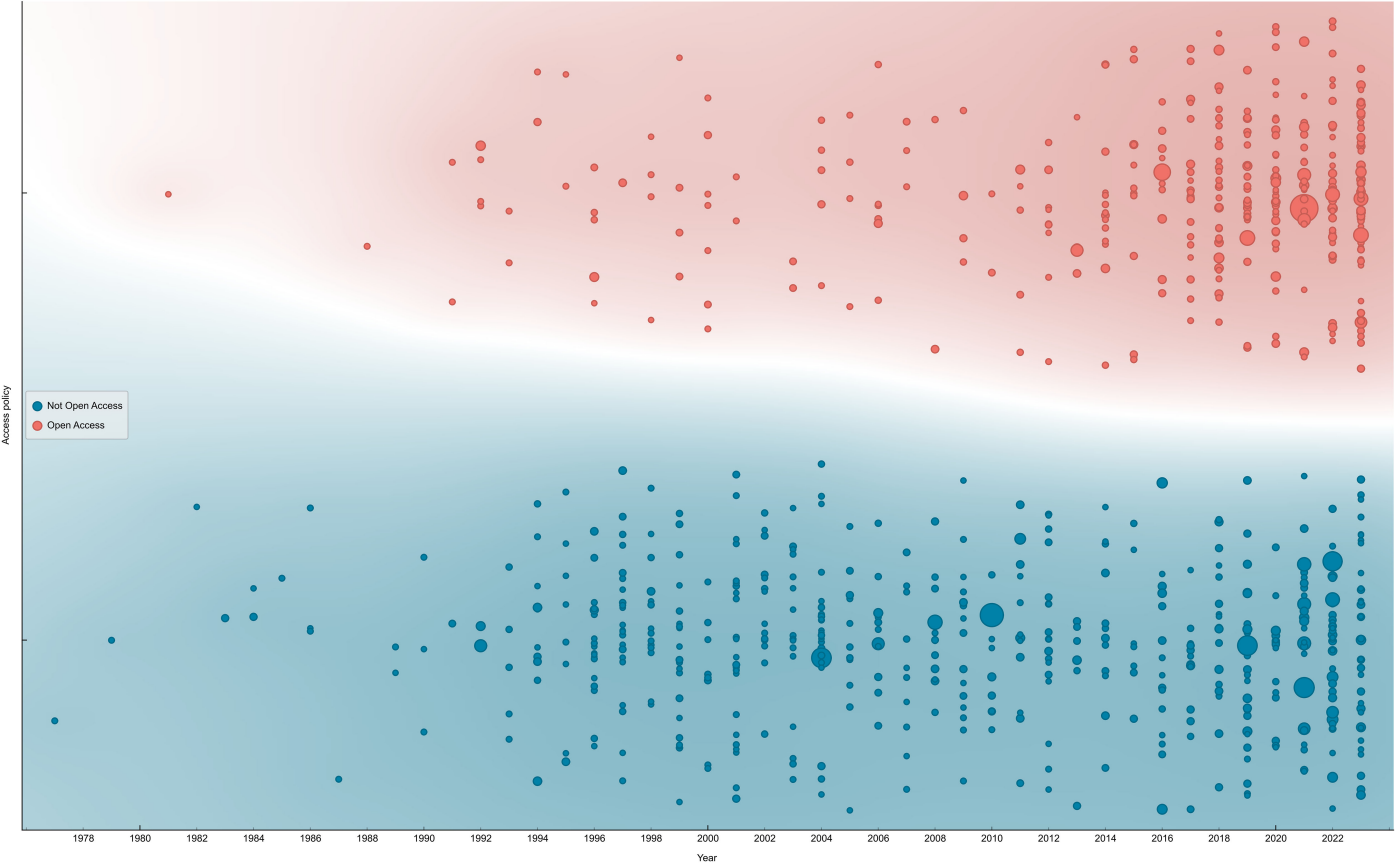
Distribution of Open Access vs Non-Open Access papers on the NFT topic over the years. The dimension of the bubbles represents the number of citations.

Another aspect we considered was the evolution of access policies for scientific papers (**Figure 13**). From 1977 to 2023, Open Access (OA) and Non-Open Access (NOA) policies represented 41% and 59%, respectively (**Figure 13**). However, this distribution results from different trends when considering various periods. By splitting the total timespan into two periods—1977-2010 and 2011-2023—the percentage of Open Access (OA) papers was 19% in the former and 56% in the latter (**Figure 13**, data not shown). This trend has become more prominent in recent years, as OA has gained increasing importance not only in scientific fields. Several studies highlight that the adoption of OA leads to a higher number of citations and helps reduce knowledge inequalities (Clayson et al., 2021; Saravudecha et al., 2023)—even when articles are embargoed for some years (Ottaviani, 2016)—and improves journal performance (though with some differences) (Asai, 2023). Furthermore, NOA papers tend to have a shorter period of attention (Wang et al., 2015), while OA papers have a citation advantage compared to NOA ones (Sotudeh and Estakhr, 2018).

**Figure 14:**
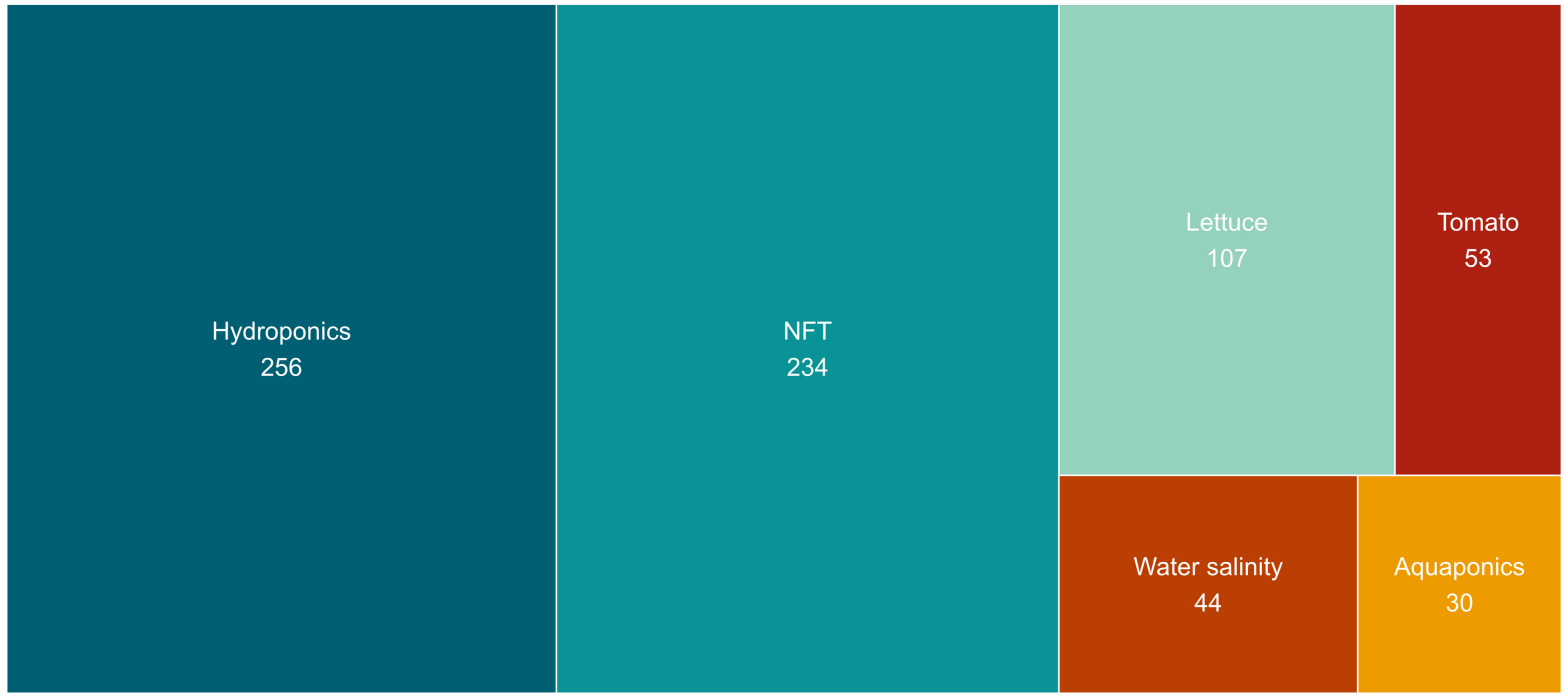
Treemap of the most common terms extracted from titles, abstracts, and keywords. Only the top six terms are displayed.

From the analysis of the most prevalent topics in the titles, abstracts, and keywords of the considered scientific publications, we found that the term "hydroponics" is present in 26% of the publications, followed by the acronym "NFT”, which appears in 24% of the publications (**Figure 14**). These two terms are closely linked to the NFT cultivation method, as NFT is a type of hydroponic cultivation (**Figure 14**).

The subsequent most commonly found terms in the scientific publications pertain to the themes addressed within them (**Figure 14**). Specifically, the third most frequently found term is "lettuce," appearing in 11% of the works, followed by "tomato" (6%), "salinity" (5%), "greenhouse," and "aquaponics" (3% each). Other terms such as "photosynthesis," "water quality," and "electrical conductivity" each appear in 1% of the works (**Figure 14**).

According to the results of this bibliometric analysis concerning the main topics covered in scientific publications on the NFT theme, the following paragraphs will analyze in more detail the principal studies conducted on "lettuce," "tomato," "salinity," and "aquaponics" using the NFT cultivation technique.

### 3.1 Key issues, emerging challenges, and opportunities in NFT systems

#### 3.1.1 Key issues

##### 3.1.1.1 Salinity tolerance

Salinity stress is a significant constraint to plant growth and productivity worldwide, particularly in soilless cultivation systems like NFT, where plants are directly exposed to saline nutrient solutions, and salinity can build up in the recirculating NS due to water evaporation and selective nutrient absorption by plants. This issue is further exacerbated in arid and semi-arid regions where water resources are scarce, and farmers often resort to using saline water for irrigation, and the accumulation of sodium chloride in the root zone can lead to osmotic stress, ion toxicity, and nutritional imbalances, ultimately affecting crop yield and quality (Santamaria et al., 2003; Munns and Tester, 2008; Saito et al., 2009; Neocleous and Savvas, 2016; Palmitessa et al., 2021).

In NFT systems, the continuous flow of nutrient solution allows for precise control of salinity levels, but the accumulation of salts over time can still pose a challenge. Several studies have investigated the effects of salinity on different crops grown in NFT systems. For instance, research has shown that salinity stress negatively impacts the yield and fruit quality of grafted watermelon (Colla et al., 2006).

Given the detrimental effects of salinity on plant production in NFT systems, researchers have proposed various mitigation strategies. One approach involves the periodic leaching of the NS to flush out accumulated salts. However, this method requires careful management to avoid nutrient wastage and environmental pollution.

Various approaches have been explored to mitigate salinity stress in NFT systems. These include:

- Selection of salt-tolerant cultivars: Certain plant species and cultivars exhibit greater tolerance to salinity stress. Identifying and cultivating these varieties can significantly improve the resilience of NFT systems to saline conditions (Munns and Tester, 2008; Neocleous and Savvas, 2016; Palmitessa et al., 2021).
- Grafting: Grafting salt-sensitive cultivars onto salt-tolerant rootstocks has shown promise in improving salinity tolerance in crops like watermelon and cucumber (Colla et al., 2006; Rouphael et al., 2008).
- Nutrient management: Adjusting the composition of the nutrient solution, such as increasing potassium levels, can mitigate the negative effects of salinity on plant growth (Dyląg et al., 2023). Potassium can help maintain ion balance and reduce sodium uptake by plants (Economakis and Daskalaki, 2003).
- Use of biostimulants: Applying biostimulants, such as glycinebetaine, can enhance plant tolerance to salinity stress. Glycinebetaine acts as an osmoprotectant, helping plants maintain cellular turgor and reduce oxidative damage under saline conditions (Uusitupa et al., 2023).
- Molecular biology and genomics approach recent advances offer promising avenues for identifying salt-tolerant genes and developing genetically modified crops. For instance, Zhang et al. (2010) identified several candidate genes associated with salinity tolerance in tomato, providing a foundation for future genetic engineering efforts.
- Advanced monitoring and control systems: technologies such as sensors and automated dosing systems can help maintain optimal NS composition, thereby mitigating the adverse effects of salinity, as highlighted by Savvas and Gruda (2018), who emphasized the potential of these technologies to enhance the resilience of NFT systems to salinity stress (**Table 1**).

**Table 1:** Main topics and related research activities on the effects of water salinity on vegetables production in NFT system.

Topic	Research activity results	References
Effects of Salinity on Plant Growth	It has been demonstrated that increased salinity leads to reduced plant growth, leaf area, fruit yield, and quality in tomatoes and cucumbers.	(Van Ieperen, 1996; Neocleous and Savvas, 2016)
	Osmotic adjustment, ion exclusion, and compartmentalization have been discussed as mechanisms for salinity tolerance, along with the potential of CRISPR/Cas9 technology for developing salt-tolerant varieties.	(Munns and Tester, 2008; Gupta and Huang, 2014)
Mitigation Strategies for Salinity Stress	The breeding of salt-tolerant varieties and the use of biostimulants to enhance root growth and nutrient uptake efficiency have been proposed.	(Ashraf and Harris, 2004; Albacete et al., 2014)
	Periodic leaching of the nutrient solution (NS) to remove accumulated salts has been suggested, requiring careful management to avoid nutrient wastage and pollution. It has been proposed to use nutrient solutions with high electrical conductivity (EC) during the night and low EC during the day.	(Van Ieperen, 1996) (Santamaria et al., 2003)
Technological Advances and Monitoring	The use of sensors and automated dosing systems has been highlighted as a means to maintain optimal nutrient solution (NS) composition and mitigate the effects of salinity stress.	(Savvas and Gruda, 2018)

##### 3.1.1.2 Nutrient management

Maintaining optimal nutrient levels in the NFT system is crucial for plant growth and development. NFT systems require careful monitoring and adjustment of nutrient concentrations to meet the specific needs of the crop. Key considerations for nutrient management in NFT systems include:

- Nutrient uptake dynamics: Different plant species and growth stages have varying nutrient requirements. Constant monitoring and adjustments of the nutrient solution are necessary to prevent deficiencies or toxicities (Endut et al., 2010; El-Nakhel et al., 2021).
- Nutrient interactions: The uptake and utilization of one nutrient can be influenced by the presence of others. For example, high levels of potassium can inhibit the uptake of magnesium. Careful balancing of nutrient ratios in the solution is essential (Bres and Weston, 1992; Ashraf and Harris, 2004; Blidariu and Grozea, 2011).
- Nutrient losses: Nutrient losses can occur through plant uptake, volatilization, and precipitation. Regular replenishment of nutrients is required to maintain optimal levels (Endut et al., 2010; Suhl et al., 2019; Tabaglio et al., 2020; El-Nakhel et al., 2021).

Researchers are exploring innovative approaches to optimize nutrient management in NFT systems, such as:

- Development of sensor-based nutrient monitoring and control systems: real-time monitoring of nutrient levels can enable automated adjustments to maintain optimal concentrations (Savvas and Gruda, 2018).
- Use of precision fertigation techniques: Precise delivery of nutrients based on plant demand can improve nutrient use efficiency and reduce waste (Suhl et al., 2019; Tabaglio et al., 2020).

##### 3.1.1.3 Oxygen availability

Adequate oxygen supply to the roots is essential for plant respiration and nutrient uptake. In NFT systems, the nutrient solution flows over the roots in a thin film, providing a continuous supply of nutrients. However, the oxygen content of the nutrient solution can fluctuate throughout the day due to factors such as temperature, plant respiration, and microbial activity, and maintaining sufficient dissolved oxygen levels can be a challenge due to (Urrestarazu et al., 2005; Park and Williams, 2024):

- High plant density: Dense planting can increase oxygen demand and limit oxygen availability in the nutrient solution, as it increases the overall oxygen demand of the root system as more roots are competing for the available oxygen in the nutrient solution (Mohammed and Sookoo, 2016).
- Temperature fluctuations: Elevated temperatures reduce oxygen solubility in water, potentially leading to oxygen stress for plants (Arancon et al., 2019).
- Organic matter accumulation: Decomposition of organic matter in the nutrient solution can deplete oxygen levels (Kano et al., 2021; Lau and Mattson, 2021).

#### 3.1.2 Emerging challenges

##### 3.1.2.1 Climate change

Climate change is a significant challenge for agriculture worldwide, and NFT systems are no exception. Rising temperatures, changes in precipitation patterns, and increased frequency of extreme weather events can all impact NFT production (Raza et al., 2019).

Some of the key challenges posed by climate change to NFT systems include:

- Water scarcity: Climate change is leading to increased water scarcity in many regions, making it more difficult to obtain high-quality water for NFT systems (Martinez-Mate et al., 2018).
- Heat stress: Elevated temperatures can cause heat stress in plants, leading to reduced growth, yield loss, and increased susceptibility to pests and diseases. Rising temperatures can affect NFT systems in various ways. Higher temperatures reduce the solubility of oxygen in water, potentially leading to oxygen stress for plant roots (Kawasaki et al., 2014). This stress can inhibit nutrient uptake and overall plant growth. Additionally, extreme heat can accelerate evaporation from the nutrient solution, necessitating more frequent replenishment and potentially increasing water usage. This factor can be particularly challenging in regions already facing water scarcity. Furthermore, heat stress may have an impact on plant physiology, by disrupting various physiological processes in plants (Topalcengiz et al., 2024).
- Increased pest and disease pressure: Warmer temperatures can favor the proliferation of certain pests and diseases, posing a greater challenge to managing them in NFT systems. This information is not from the sources and may need to be independently verified.

##### 3.1.2.2 Microbial contamination

NFT systems provide a favorable environment for the growth of microorganisms, some of which can be pathogenic to plants or humans. Microbial contamination can pose significant challenges to food safety and crop production, as the warm, humid conditions and the presence of nutrients in the NFT solution can facilitate rapid microbial growth.

Key concerns related to microbial contamination in NFT systems include:

- Microbial hazards and risks: while soilless systems can offer advantages over traditional soil-based cultivation, they are not exempt from microbial contamination. The unique environment of soilless systems, particularly the nutrient-rich solution used in NFT, can create conditions conducive to microbial growth. In their article, Topalcengiz et al. (2024) gave a broad overview of microbial hazards and risks inherent in indoor soilless production of leafy greens, encompassing NFT systems, and the Authors emphasizes the importance of recognizing and mitigating these risks to ensure food safety.
- NFT as a potential solution for zoosporic pathogens: By eliminating soil, a common reservoir for zoosporic pathogens, NFT can reduce the risk of infection. This benefit underscores the importance of maintaining strict hygiene and sanitation practices to prevent the introduction and spread of these pathogens within the NFT system itself. Stanghellini and Rasmussen (1994) explored the potential of hydroponics, including NFT, as a solution for managing zoosporic pathogens, a type of waterborne disease-causing organism. The Authors suggested that the controlled environment and the absence of soil in hydroponic systems can limit the spread and impact of these pathogens. However, great attention should be posed on microbial safety in soilless systems, as discussed below.
- Microbial safety in soilless systems: the soilless systems are vulnerable to microbial proliferation due to the absence of soil's natural buffering capacity. In this regard, NFT in particular may be affected, since such system does not have any buffer capacity. As reported by Dorick et al. (2021), the quality of the source water used for preparing nutrient solutions is crucial for microbial safety. Contaminated source water can introduce pathogens and spoilage microorganisms into the system. The Authors highlighted that regular monitoring and treatment of source water are essential to minimize the risk of introducing contaminants into the NFT system. This could include filtration, disinfection, or other appropriate treatment methods.

##### 3.1.2.3 Sustainability

NFT, while offering numerous advantages, face growing scrutiny regarding their environmental sustainability. Water usage, energy consumption, and waste management are key areas of concern.

- Water usage: although NFT systems generally use less water than traditional agriculture, they still require significant amounts of water, especially in arid and semi-arid regions (Pius Awhari et al., 2024). This demand stems from the need to maintain the nutrient solution and compensate for water loss through evapotranspiration (Fussy and Papenbrock, 2022). Hydroponically grown lettuce, for example, used about 10 per cent of the water compared to open agriculturally grown lettuce (Treftz and Omaye, 2016). The continuous flow of nutrient solution in NFT systems, while ensuring plants have access to all the necessary nutrients, can lead to higher water consumption compared to other hydroponic methods (Uusitupa et al., 2023).
- Energy consumption: NFT systems rely on pumps, aeration systems, and other equipment that consume energy (Pius Awhari et al., 2024). The sources specifically mention that energy consumption for desalination in regions with limited freshwater resources poses a challenge. Desalinated seawater, while offering a potential solution for water scarcity, requires high energy input, contributing to greenhouse gas emissions. Energy consumption and greenhouse gas emissions increase linearly when conventional water resources are replaced by desalinated seawater. Using renewable energy sources could mitigate this impact, reducing emissions (Martinez-Mate et al., 2018).
- Water management in NFT systems: several authors (Savvas et al., 2002; Martinez-Mate et al., 2018; De Wrachien et al., 2021; Pius Awhari et al., 2024) have emphasized the importance of careful water management in NFT systems to ensure sustainability, with particular reference to:

o Salinity accumulation in the recycled nutrient solution is a major challenge, especially when using desalinated seawater (Savvas et al., 2002; Martinez-Mate et al., 2018; Suwaileh et al., 2020). This accumulation can negatively impact plant growth and necessitate the disposal and replacement of the nutrient solution, leading to water and nutrient waste (Savvas et al., 2002).

o Nitrate accumulation: The continuous access to nutrients in NFT systems can lead to excessive nitrate uptake by plants (Uusitupa et al., 2023). Various approaches to mitigate nitrate accumulation are discussed in the sources, including using chloride in the nutrient solution and optimizing nitrogen fertilization (Voutsinos et al., 2021).

o Maintaining water quality in NFT systems is crucial for optimal plant growth and preventing the spread of diseases (Park and Williams, 2024). The sources highlight the need for careful monitoring and management of pH, dissolved oxygen, and nutrient levels (Park and Williams, 2024). Various techniques, including UV sterilization, ozonation, and biofiltration, can be employed to prevent infections in the nutrient solution (Velazquez-Gonzalez et al., 2022).

#### 3.1.3 Opportunities

##### 3.1.3.1 Vertical farming

NFT systems are well-suited for vertical farming due to their ability to grow crops in a limited space without soil. Vertical farming, a method of growing crops in vertically stacked layers, is gaining popularity, especially in urban environments. This technique offers several advantages over traditional farming, including:

- Higher yields: NFT systems can achieve higher yields than traditional agriculture due to their precise control over environmental factors and nutrient delivery (Martinez-Mate et al., 2018).
- Reduced water usage: NFT systems can significantly reduce water consumption compared to conventional agriculture. This is particularly important in urban areas where water resources may be limited (Barbosa et al., 2015)
- Year-round production: Vertical farms with NFT systems can operate year-round, independent of weather conditions. This allows for a consistent supply of fresh produce, regardless of the season.
- Reduced transportation costs: Vertical farms located in or near urban areas can minimize transportation costs associated with delivering produce to consumers. This can also enhance food security and reduce the carbon footprint associated with food transportation (Treftz and Omaye, 2016).

##### 3.1.3.2 Biofortification

NFT systems offer precise control over nutrient delivery, making them ideal for biofortification, the process of enhancing the nutritional value of crops. By manipulating the nutrient composition of the solution, specific minerals and vitamins can be enriched in the edible portions of plants, like:

- Iodine biofortification: Iodine deficiency is a global health concern, and biofortified crops can contribute to improving iodine intake. Adding iodine to the nutrient solution can increase iodine levels in crops, e.g. in lettuce (Smoleń et al., 2014, 2016, 2019).
- Selenium biofortification: Selenium is an essential micronutrient with antioxidant properties. Selenium levels in lettuce can be enhanced through biofortification in NFT systems (Kowalska et al., 2020).
- Iron biofortification: Iron deficiency is another widespread nutritional problem. NFT systems can be used to biofortify crops with iron, increasing their iron content (Giordano et al., 2019).

##### 3.1.3.3 Integration with aquaponics

Aquaponics is a sustainable system that combines aquaculture (raising aquatic animals like fish) with hydroponics (growing plants in water without soil). Aquaponics leverages the natural nutrient cycle, where fish waste provides a source of nutrients for plants, and the plants in turn help purify the water for the fish (Alias et al., 2022; Kumar and Singh, 2023). This creates a closed-loop system that is both environmentally friendly and efficient (Blidariu and Grozea, 2011). When combined with the NFT system, which delivers a thin film of NS directly to plant roots, aquaponic setups can achieve efficient nutrient delivery while minimizing water usage (Alias et al., 2022). This approach enhances nutrient cycling and promotes sustainable agricultural practices (Blidariu and Grozea, 2011).

The system usually consists of a fish tank, water pump and filtration system and a settling tank (Alias et al., 2022), in addition to an aeration system (Al-Zahrani et al., 2023).

The most farmed fish species are Nile Tilapia (*Oreochromis niloticus*), Empurau (*Tor tambroides*), Hoven Carp (*Leptobarbus hoevenii*) and Silver Barb (*Barbonymus gonionotus*) (Alias et al., 2022).

The benefits of integrating NFT with aquaponics include:

- Enhanced nutrient utilization: NFT's efficient nutrient delivery mechanism ensures that the nutrients from fish waste are effectively absorbed by plants. Researchers have explored various strategies to optimize nutrient availability and uptake, considering the unique dynamics of aquaponic ecosystems (Lennard and Leonard, 2006). A study by Endut et al. (2010) focused on optimizing nutrient ratios in aquaponic NFT systems to enhance plant growth. By adjusting the composition of fish feed and monitoring nutrient levels in the water, they achieved improvements in yields and nutrient uptake in lettuce plants. This approach highlights the importance of balancing fish nutrition with plant requirements to maintain system productivity. The Authors calculated the ratio of fish to plant production to balance nutrient generation from fish with nutrient removal by plants, and the optimum ratio was 15–42 gram of fish feed/m^2^ of plant growing area (Blidariu and Grozea, 2011) and discovered that while increasing the number of plants initially enhanced nutrient removal, there was a limit to this effect. Beyond a certain plant-to-fish ratio, adding more plants did not significantly improve nutrient removal. This suggests that there is an optimal ratio beyond which further increases in plant density don't translate to proportionally higher nutrient uptake.
- Improved water quality: plants in the NFT system act as a biofilter, removing excess nutrients from the water and maintaining a healthy environment for the fish (Al-Zahrani et al., 2023).
- Increased production: the combination of aquaponics and NFT can lead to increased yields of both fish and plants compared to traditional methods. Delaide et al. (2016) investigated the performance of lettuce plants grown in an aquaponic NFT system compared to conventional hydroponics. They found that plants in the aquaponic NFT setup exhibited similar or even superior growth rates and nutrient uptake compared to those in the hydroponic system.
- Space efficiency: NFT systems can be designed vertically, making them ideal for space-constrained environments, further enhancing the space-saving benefits of aquaponics (Alias et al., 2022).

Sustainability: the integrated system reduces water usage, minimizes waste, and avoids the need for synthetic fertilizers, contributing to a more sustainable food production model. Blidariu and Grozea (2011) explored the use of alternative fish species and plant species to increase system diversity and resilience. By incorporating multiple fish species with different ecological niches and selecting plant varieties with varying nutrient requirements, they created more robust aquaponic NFT systems capable of adapting to changing conditions. The Authors focused on the interdependence of the fish and plant components and the potential for disruptions in the system's balance, since aquaponics systems rely on a delicate equilibrium between fish waste production and nutrient uptake by plants. This interdependence may create vulnerabilities as any disruption in one component can cascade through the entire system in different ways like nutrient fluctuations (factors like fish stocking density, feed composition, and fish health can significantly influence nutrient levels in the water, potentially leading to imbalances) and disease susceptibility (both fish and plants are susceptible to other pathogens) (Al-Zahrani et al., 2023). On the contrary, the increased diversity and resilience furnish some benefits such as enhanced stability, Improved water quality, Disease resistance and, finally, a greater adaptability (Blidariu and Grozea, 2011).

A recent review by Topalcengiz et al. (2024) outlined emerging trends and future directions in aquaponics research, including the integration of advanced monitoring and control systems, the development of novel aquaponic designs, and the exploration of alternative fish and plant species. These efforts aim to enhance the sustainability, productivity, and scalability of aquaponic NFT systems for future agricultural applications.

The integration of aquaponics and NFT presents a promising approach for sustainable plant production, leveraging the synergies between aquaculture and hydroponics. Recent research has demonstrated the potential of aquaponic NFT systems to achieve efficient nutrient delivery, enhance system resilience, and optimize resource utilization. By addressing challenges and embracing technological advancements, aquaponic NFT systems can contribute to the advancement of sustainable agriculture and food security (**Table 2**).

**Table 2:** Main topics and related research activities on hybrids Aquaponic and NFT systems.

Topic	Research activity results	References
Efficiency and Nutrient Management	The efficient nutrient delivery and sustainable agricultural practices through nutrient cycling in aquaponic NFT systems have been highlighted.	(Blidariu and Grozea, 2011; Alias et al., 2022)
	Similar or superior plant growth in aquaponic NFT systems compared to conventional hydroponics has been demonstrated.	(Delaide et al., 2016)
	The importance of effective nutrient management for maximizing plant growth and ensuring system health has been emphasized.	(Lennard and Leonard, 2006)
	Improved yields and nutrient uptake in lettuce have been achieved by optimizing nutrient ratios and balancing fish and plant requirements.	(Endut et al., 2010)
Resilience and System Diversity	The use of alternative fish species and plant varieties to increase system diversity and resilience has been explored.	(Blidariu and Grozea, 2011)
Technological Innovations and Future Directions	Emerging trends have been outlined, including advanced monitoring and control systems, novel designs, and alternative species, aimed at enhancing system sustainability, productivity, and scalability.	(Topalcengiz et al., 2024)

### 3.2 Application of NFT to commercially important vegetable species

NFT systems may be applied for the actual cultivation of vegetables. In the following paragraphs we report two examples of NFT cultivation for lettuce and tomato crops using the NFT system.

#### 3.2.1 Leaf nitrate accumulation and biofortification in lettuce using the NFT system

The term "lettuce" appears in 107 papers within the considered databases (**Figure 14**). The NFT cultivation method has been widely utilized to study how the composition of the NS affects the concentration of nutrients, particularly nitrates, in lettuce. Bres and Weston (1992) studied how varying the potassium concentration from 150 to 225 mg/L and adjusting the pH of the NS from 5.0 to 6.5 do not significantly alter the concentration of nutrients in lettuce leaves. More recently, with the development of Light-Emitting Diode (LED) technology, the application of LEDs has been combined with the NFT cultivation technique. In a study conducted by Soufi et al. (2023), it was found that the application of red and blue artificial light spectra, together with NS management based on volume replenishment according to plant uptake, increases the concentration of macro and micronutrients in leaf tissues, reduces nitrate accumulation, and promotes the photosynthetic activity of the plant.

Conversely, Tabaglio et al. (2020) demonstrated that to reduce nitrate concentration in lettuce leaves, it is necessary to suspend fertilizer application two to four days before harvest. However, this technique achieves a greater reduction of nitrates in the spring cycle compared to the summer cycle, probably due to the higher intensity of radiation present during the summer. Furthermore, these climatic influences on nitrate accumulation in lettuce were also observed in a study by Urlić et al. (2017), which showed that reducing the NO_3_^-^:NH_4_^+^ ratio decreases nitrate concentration in lettuce leaves.

A recent study explored an innovative method to reduce nitrate concentration in lettuce leaves: the application of glycinebetaine (GB), a quaternary ammonium compound that is endogenously accumulated in many higher plants (though not in lettuce) and is a natural part of the human diet. Uusitupa et al. (2023) demonstrated that cultivating lettuce in NFT and adding GB to the NS at concentrations up to 10 mM reduces nitrate concentration in lettuce by up to 29% while increasing the concentration of the amino acid fraction. Further research has been conducted using the NFT technique to study nitrate accumulation in lettuce (Dalastra et al., 2020; Djidonou and Leskovar, 2019; Gunes et al., 1994; Sularz et al., 2020; Urrestarazu et al., 1998; Valverde et al., 2009). The NFT system, due to the absence of a substrate, is particularly well-suited for these types of studies, as working without a substrate eliminates its interference with nutrient absorption and accumulation in the root environment.

Another research topic associated with the NFT cropping system is biofortification, which involves the administration of mineral elements (e.g., iodine) in the NS, especially in lettuce (Sularz et al., 2020). Studies conducted by Kowalska et al. (2020) and Smoleń et al. (2019, 2017, 2015) have demonstrated that administering salicylic acid in the nutrient solution can influence, at certain doses, the accumulation of iodine and selenium in the leaf tissues of lettuce. Regarding iodine and selenium biofortification, a study by Smoleń et al. (2014) showed that the transport of iodine and selenium in lettuce, from the leaves to the roots, occurs via the phloem; thus, root vegetables may be biofortified with these elements through both root administration and foliar application. In another study, Dyląg et al. (2023) demonstrated that iodine application through the NS can occur not only in mineral form (almost always as potassium iodate) but also in organic form (as iodoquinolines). However, unlike the mineral form, the organic form exhibits biostimulant activity on lettuce, leading to increased yield and greater stress resistance.

Besides iodine and selenium biofortification, iron biofortification of lettuce has also been studied using the NFT cultivation technique (Giordano et al., 2019). The regulation of nitrate content in leaves and biofortification has been the main research focus regarding the application of NFT for lettuce cultivation. However, there are also other topics that are addressed less frequently, such as the application of different cultivation strategies (Majid et al., 2021), the effect of varying NS composition on plant yield and quality (El-Nakhel et al., 2021), and the energy and environmental sustainability of cultivation (Martinez-Mate et al., 2018).

The use of lettuce as a “model crop” to study leaf nitrate accumulation and biofortification in NFT systems represents a valuable research approach for improving the nutritional quality and safety of leafy vegetables. By understanding the factors influencing nitrate accumulation, implementing biofortification strategies, optimizing environmental conditions, and leveraging genetic approaches, researchers can contribute to the development of healthier and more nutritious lettuce varieties (**Table 3**).

**Table 3:** Main topics and related research activities on lettuce cultivated in NFT.

Topic	Research activity results	References
Reduction of nitrate accumulation in lettuce leaves	Varying potassium concentration and adjusting pH in the nutrient solution (NS) do not significantly alter nutrient concentration.	(Bres and Weston, 1992)
	The use of red and blue LED light spectra with nutrient solution (NS) management increases nutrient concentration, reduces nitrate levels, and enhances photosynthetic activity.	(Soufi et al., 2023)
	Suspending fertilizer application 2-4 days before harvest reduces nitrate concentration, with greater effectiveness in spring.	(Tabaglio et al., 2020)
	Reducing NO_3_^-^: NH_4_^+^ ratio decreases nitrate concentration, with optimal nitrogen use efficiency achieved at 0-50% ammonium ion of total nitrogen.	(Urlić et al., 2017)
	Adding glycinebetaine (GB) to the NS at concentrations up to 10 mM reduces nitrate concentration by 29% and increases amino acid concentration.	(Uusitupa et al., 2023)
Biofortification	The administration of salicylic acid in the nutrient solution (NS) influences iodine and selenium accumulation in leaf tissues.	(Smoleń et al., 2019; Kowalska et al., 2020, 2017, 2015)
	Iodine and selenium are transported from leaves to roots via the phloem, which can enrich root vegetables.	(Smoleń et al., 2014)
	Iodine application in organic form (iodoquinolines) acts as a biostimulant, increasing yield and stress resistance.	(Dyląg et al., 2023)
	Iron biofortification of lettuce using the NFT system.	(Giordano et al., 2019)
Other NFT-related research topics	Various cultivation strategies, the effect of NS composition on yield and quality, and the energy and environmental sustainability of cultivation are important considerations.	(Martinez-Mate et al., 2018; El-Nakhel et al., 2021; Majid et al., 2021)

#### 3.2.2 Harnessing the potential of tomato cultivation with NFT system

Tomatoes are the most important vegetables crop, and they are widely consumed and cultivated globally (Palmitessa et al., 2020). Based on the databases analyzed in this review, the word "tomato" appears in the titles, abstracts, and/or keywords of 54 documents. Fresh tomatoes are long-cycle vegetable crops that do not adapt perfectly to the NFT cultivation system, as extensive root growth over time may restrict the flow of the NS within the system, leading to reduced supply of water, nutrients, and oxygen. Consequently, one of the most frequently studied topics in tomato cultivation using NFT systems is the concentration of oxygen in the NS.

In a study by Gislerød and Kempton (1983), the authors demonstrated that cultivating tomatoes and cucumbers in NFT affects growth, leaf area, productivity, and root development. They also highlighted that oxygen concentrations in the NS, ranging from 1 to 3 mg/L, reduce plant growth, although such concentrations do not cause highly adverse effects on cultivation. Conversely, Suhl et al. (2019) reported that oxygen concentrations below 5 mg/L may result in stress symptoms and stunted growth.

The sensitivity of crops to oxygen levels in the NS primarily depends on the cultivation system and the volume of NS in contact with the roots. When a large volume of NS is available to the roots, plants can tolerate lower oxygen concentrations without exhibiting stress symptoms. To mitigate potential stress from reduced dissolved oxygen in the NS, López-Pozos et al. (2011) demonstrated that increasing the gutter slope from 2% to 4% decreases dissolved oxygen depletion in the NS and increases tomato yield. Additionally, Lycoskoufis and Mavrogianopoulos (2020) designed a system called the "Nutrient Drip Technique" (NDT), in which the NS is delivered via drippers at multiple points along the channel. The drainage is collected at several points along the same channel to enhance oxygenation.

In their study on tomatoes, Lycoskoufis and Mavrogianopoulos (2020) demonstrated that the Nutrient Drip Technique (NDT) system maintains a higher concentration of dissolved oxygen in the NS compared to the traditional NFT system. Additionally, the greater flow of NS circulating in the system allows for an increased channel length relative to the NFT system, without causing significant differences in the growth of plants located at the upstream and downstream ends of the channel.

Another modification to the NFT cultivation system was proposed by Urrestarazu et al. (2005), called the "New Growing System" (NGS). This system consists of five plastic layers with multiple holes, featuring a dripper placed every 0.5 m above the first plastic layer. Each dripper supplies the NS, which flows from the second layer over the others to the end. Using this cultivation system, the concentration of dissolved oxygen in the NS decreased from 7.12 to 6.65 mg/L over a distance of 20 meters, whereas, in the traditional NFT system, it dropped from 6.2 to 2.9 mg/L (Urrestarazu et al., 2005).

However, the study of dissolved oxygen in the NS is only one of the topics investigated through research activities involving NFT for tomato cultivation. Other studies conducted using NFT aim to: (i) test the effects of salinity on the quality and quantity of produced berries (Van Ieperen, 1996; Saito et al., 2009); (ii) understand how different nitrogen-to-potassium ratios affect tomato growth and production (Economakis and Daskalaki, 2003); (iii) investigate the effects of basal heating on photosynthetic activity and tomato productivity (Kawasaki et al., 2014); (iv) estimating the nutrient uptake concentrations, thanks to the absence of a substrate that does not interfere with absorption (Savvas et al., 2017); and (v) explain the physiological mechanisms related to the uptake of heavy metals such as copper (Liao et al., 2000) or nickel and cadmium (Degryse and Smolders, 2012).

The utilization of tomato plants in NFT represents a promising approach to enhance yield, quality, and resource use efficiency in tomato cultivation. By leveraging precise nutrient delivery, environmental control, and genetic diversity, researchers are unlocking the full potential of NFT-based tomato production. Investigating more sustainable cultivation techniques is essential to reduce the impact of agricultural activity on the ecosystem. Currently, tomatoes, along with cucumbers and peppers, are primarily grown using soilless cultivation systems on rock wool slabs. This growing medium is often disposed of after one cultivation cycle and is not always recyclable, resulting in up to 150 m³ of rock wool waste per hectare produced annually. Furthermore, an average primary energy demand of 275 kWh is required to produce one cubic meter of rock wool, releasing 167 kg of CO2 into the environment (Dannehl et al., 2015). Continued innovation and collaboration across disciplines will further advance this field, ensuring a sustainable and resilient future for tomato growers worldwide (**Table 4**).

**Table 4:** Main topics and related research activities on tomato cultivated in NFT system.

Topic	Research activity results	References
Oxygen content in the NS	Oxygen concentrations of 1-3 mg/L have been shown to reduce plant growth but are not very severe.	(Gislerød and Kempton, 1983)
	Concentrations below 5 mg/L can cause stress and stunted growth.	(Suhl et al., 2019)
	Increasing the gutter slope from 2% to 4% decreases dissolved oxygen depletion and increases yield.	(López-Pozos et al., 2011)
	The "Nutrient Drip Technique" (NDT) has been designed to maintain higher dissolved oxygen levels and allow for longer channels.	(Lycoskoufis and Mavrogianopoulos, 2020)
	The “New Growing System” (NGS) has been developed, which shows reduced oxygen depletion compared to traditional NFT.	(Urrestarazu et al., 2005)
NS composition	It has been tested how salinity affects the quality and quantity of tomato berries.	(Van Ieperen, 1996; Saito et al., 2009)
	It has been studied how different nitrogen to potassium ratios affect tomato growth and production.	(Economakis and Daskalaki, 2003)
Heavy metal and abiotic stresses	It has been investigated how basal heating affects photosynthetic activity and productivity.	(Kawasaki et al., 2014)
	The uptake mechanisms of heavy metals like copper, nickel, and cadmium have been researched.	(Liao et al., 2000; Degryse and Smolders, 2012)

## 4 Conclusion

In this review, we conducted a bibliometric analysis of 774 scientific documents on NFT, highlighting the evolution and increasing interest in this cultivation method from 1977 to 2023. The analysis revealed a growing trend in NFT-related publications, marked by significant peaks in productivity and a notable shift from conference papers to peer-reviewed journal articles and reviews. *Acta Horticulturae* emerged as the leading journal in this field, underscoring the importance of conference contributions in the early years of NFT research.

Lettuce and tomato have been the primary focus of NFT studies, reflecting the technique's applicability to a wide range of crops. Research on lettuce has concentrated on nitrate accumulation and biofortification, demonstrating the potential to enhance nutritional quality and safety. Meanwhile, studies on tomatoes have highlighted the challenges associated with oxygen concentration in the NS, with innovations such as the "Nutrient Drip Technique" (NDT) and "New Growing System" (NGS) showing promise in mitigating these issues.

However, it should be noted that in commercial systems, NFT commonly used for the cultivation of smaller species (such as lettuce and rocket) rather than tomatoes, as the latter is a long-term crop whose root mass would eventually fill the channels.

The impact of water salinity on plant growth in NFT systems has also been extensively examined, revealing the need for advanced salinity tolerance strategies and optimized nutrient management. Furthermore, the integration of aquaponics and NFT systems has shown potential for sustainable and efficient crop production, although challenges such as nutrient imbalances and disease management persist.

Overall, this bibliometric review underscores the growing importance and versatility of NFT in soilless cultivation. Continued research and innovation are essential to address existing challenges and fully harness the potential of NFT for sustainable agriculture. Future studies should focus on refining nutrient delivery systems, enhancing environmental control, and exploring new crop varieties to further improve the efficiency and resilience of NFT cultivation.
